# An IL-5 Single-Nucleotide Polymorphism Influences Neuroinflammation and Prospective Disease Activity in Multiple Sclerosis

**DOI:** 10.3390/ijms25169108

**Published:** 2024-08-22

**Authors:** Ettore Dolcetti, Fabio Buttari, Antonio Bruno, Federica Azzolini, Luana Gilio, Angela Borrelli, Veronica Di Caprio, Gianluca Lauritano, Giovanni Galifi, Stefano Gambardella, Rosangela Ferese, Emiliano Giardina, Valentina Rovella, Roberto Furlan, Annamaria Finardi, Alessandra Musella, Sara Balletta, Georgia Mandolesi, Diego Centonze, Mario Stampanoni Bassi

**Affiliations:** 1 Neurology Unit, IRCCS Neuromed, Via Atinense 18, 86077 Pozzilli, Italy; dolcettiettore@gmail.com (E.D.); fabio.buttari@gmail.com (F.B.); brunoa.neuro@gmail.com (A.B.); federica.azzolini@gmail.com (F.A.); gilio.luana@gmail.com (L.G.); borrelliangela8790@gmail.com (A.B.); veronicadicaprio91@gmail.com (V.D.C.); gianlucalaurita@gmail.com (G.L.); giovalifi@gmail.com (G.G.); stefano.gambardella@neuromed.it (S.G.); ferese.rosangela@gmail.com (R.F.); balletta.sara@gmail.com (S.B.); m.stampanonibassi@gmail.com (M.S.B.); 2 PhD Program in Neuroscience, Department of Systems Medicine, University of Rome Tor Vergata, Via Montpellier 1, 00133 Rome, Italy; 3 Department of Systems Medicine, University of Rome Tor Vergata, Via Montpellier 1, 00133 Rome, Italy; rovella@med.uniroma2.it; 4 Faculty of Psychology, Uninettuno Telematic International University, 00186 Rome, Italy; 5 Department of Biomolecular Sciences, University of Urbino “Carlo Bo”, Via I Maggetti, 26, 61029 Urbino, Italy; 6 Genomic Medicine Laboratory, IRCCS Fondazione Santa Lucia, Via Ardeatina, 00179 Rome, Italy; emiliano.giardina@uniroma2.it; 7 Department of Biomedicine and Prevention, University of Rome Tor Vergata, Via Montpellier 1, 00133 Rome, Italy; 8 Clinical Neuroimmunology Unit, Institute of Experimental Neurology (INSpe), Division of Neuroscience, IRCCS San Raffaele Scientific Institute, Via Olgettina, 20132 Milan, Italy; furlan.roberto@hsr.it; 9 Faculty of Medicine and Surgery, Vita e Salute San Raffaele University, Via Olgettina, 20132 Milan, Italy; finardi.annamaria@hsr.it; 10 Synaptic Immunopathology Lab, IRCCS San Raffaele Roma, Via della Pisana, 235, 00163 Rome, Italy; alessandra.musella@uniroma5.it (A.M.); georgia.mandolesi@uniroma5.it (G.M.); 11 Department of Human Sciences and Quality of Life Promotion, University of Rome San Raffaele, Via della Pisana, 235, 00163 Rome, Italy

**Keywords:** IL-5, rs2069812, single-nucleotide polymorphism, multiple sclerosis, neuroinflammation

## Abstract

(1) Multiple sclerosis (MS) is identified by a complex interaction between central inflammation and neurodegeneration. Genetic individual variability could play a significative role in clinical presentation. The interleukin-5 (IL-5) rs2069812 single-nucleotide polymorphism (SNP) seems to define the clinical course of Th2 autoimmune diseases, while its role in MS has never been investigated. (2) In a group of 230 patients diagnosed with relapsing–remitting MS (RR-MS) or progressive MS (P-MS) and controls (IC), rs2069812 polymorphism, cerebrospinal fluid (CSF) levels of inflammatory mediators, and clinical and demographic characteristics were determined. In RR-MS patients, No Evidence of Disease Activity (NEDA-3) at three years of follow-up was detected. (3) We identified higher levels of proinflammatory cytokines, particularly IL-2 (median [IQR], RR-MS = 0.2 [0–0.7]; P-MS = 0.1 [0–1.6]; IC = 0.1 [0.0–0.1]; *p* < 0.005), IL-6 (RR-MS = 0.9 [0.3–2.3]; P-MS = 0.8 [0.1–2.7]; IC = 0.1 [0.0–0.5]; *p* < 0.005), IL-12 (RR-MS = 0.5 [0–1.1]; P-MS = 0.5 [0–1.1]; IC = 0.0 [0.0–0.3]; *p* < 0.005), and GM-CSF (RR-MS = 15.6 [4.8–26.4]; P-MS = 14 [3.3–29.7]; IC = 8.9 [4.7–11.7]; *p* < 0.005) in MS patients compared with IC. Conversely, anti-inflammatory cytokines, specifically IL-5 (RR-MS = 0.65 [0–2.4]; P-MS = 0.1 [0–0.8]; IC = 1.7 [0.6–2.8]; *p* < 0.005) and IL-1ra (RR-MS = 14.7 [4.9–26.4]; P-MS = 13.1 [4.7–22.2]; IC = 27.8 [17.7–37.6]; *p* < 0.005) were higher in controls. According to rs2069812, in MS patients, the T-allele was associated with higher concentrations of proinflammatory mediators (IL-2, CT/TT = 0.2 [0.0–2.0]; CC = 0.1 [0.0–0.4], *p =* 0.015; IL-6, CT/TT = 1.2 [0.4–3.2] vs. CC = 0.7 [0.1–1.7], *p =* 0.007; IL-15, CT/TT = 0.1 [0.0–9.5] vs. CC = 0.0 [0.0–0.1], *p =* 0.019; and GM-CSF, CT/TT = 0.1 [0.0–0.6] vs. CC = 0.05 [0.0–0.1], *p* < 0.001), and CC was associated with anti-inflammatory mediators (IL-5, CT/TT = 0.03 [0.0–1.9] vs. CC = 1.28 [0.0–2.7], *p =* 0.001; IL-1ra, CT/TT = 12.1 [4.1–25.9] vs. CC = 18.1 [12.1–26.9], *p =* 0.006). We found the same differences in RR-MS patients (IL-2, T-allele median [IQR] = 0.3 [0.0–2.0] vs. C-allele, median [IQR] = 0.04 [0.0–0.3]; *p =* 0.005; IL-6, T-allele, median [IQR] = 1.3 [0.4–3.3] vs. C-allele, median [IQR] = 0.6 [0.03–1.5]; *p =* 0.001; IL-15, T-allele, median [IQR] = 0.1 [0.0–9.5] vs. C-allele, median [IQR] = 0.0 [0.0–0.1]; *p =* 0.008; GM-CSF, T-allele, median [IQR] = 0.1 [0.0–97.9] vs. C-allele, median [IQR] = 0.0 [0.0–0.001]; *p* < 0.001; IL-5, T-allele, median [IQR] = 0.02 [0.0–2.2] vs. C-allele, median [IQR] = 1.5 [0.0–2.9]; *p =* 0.016; and IL-1ra, T-allele, median [IQR] = 12.1 [4.3–26.4] vs. C-allele, median [IQR] = 18.5 [12.7–28.3]; *p =* 0.006) but not in P-MS, except for IL-5 (T-allele, median [IQR] = 0.1 [0–0.23] vs. C-allele, median [IQR] = 0.6 [0.0–2.5]; *p =* 0.022). Finally, we identified an association between CC in RR-MS patients and NEDA-3 after three years of follow-up (*p =* 0.007). (4) We describe, for the first time, the role of an SNP of the IL-5 gene in regulating central neuroinflammation and influencing clinical course in MS patients.

## 1. Introduction

Multiple sclerosis (MS), a chronic autoimmune disease of the central nervous system (CNS) characterized by inflammation, demyelination, and neurodegeneration, occurs with a wide variety of symptoms and clinical presentations [1]. Immune system dysregulation in MS results from a complex interaction between genetic predisposition and environmental influences [2].

Individual variability in genes for cytokines and their receptors may play a role in MS clinical course [3,4]. The balance between pro- and anti-inflammatory mediators regulates immune system activation, critically influencing prospective disease activity and disability in MS [5,6]. It has been shown that single-nucleotide polymorphisms (SNPs) of genes encoding for some major proinflammatory cytokines, such as interleukin (IL)-6 and IL-8, may influence disease activity and progression in MS [7,8]. SNPs influencing the expression and activity of anti-inflammatory mediators could contribute to clinical variability in MS as suggested for other inflammatory diseases [9,10]. However, the role of SNPs of genes for anti-inflammatory cytokines in MS has been poorly explored.

The rs2069812 SNP of the IL-5 gene has gained particular interest in different neuroinflammatory disorders [11]. This SNP has been implicated in the variability of IL-5 expression and function, and thereby may also influence the CNS inflammatory environment in MS [12]. IL-5 is an anti-inflammatory cytokine traditionally known for its role in the growth, differentiation, and activation of eosinophils, which are pivotal in allergic responses and asthma [13]. In MS, IL-5 has been associated with immunomodulatory effects, promoting the production of anti-inflammatory cytokines and the expression of regulatory T cells (Tregs), crucial for maintaining immune tolerance and preventing autoimmunity. Furthermore, higher levels of this cytokine have been associated with a lower number of relapses in patients with MS [14].

To explore the role of the rs2069812 SNP of the IL-5 gene in MS, we analyzed the associations between rs2069812 SNP, neuroinflammation, and clinical characteristics, in a group of newly diagnosed MS patients and in a group of control patients.

## 2. Results

### 2.1. Clinical and Demographic Characteristics of Study Population

Clinical and demographic characteristics of MS patients and inflammatory controls (IC) are shown in Table 1.

No significant differences were found in sex distribution between RR-MS, P-MS and IC groups. Age at the time of diagnosis was significantly higher in the P-MS group than in both the RR-MS group (age at LP, median [IQR]: RR-MS = 33.9 [25.7–44.0]; *p* < 0.001) and control group (age at LP, median [IQR]: controls = 41.8 [32.3–49.7]; P-MS = 50.3 [45.6–56.8]; *p* < 0.001). In addition, significant differences were observed between RR- and P-MS patients in disease duration (RR-MS, median [IQR] = 5 [1.1–24.3]; P-MS, median [IQR] = 24.6 [11.8–72.6]; *p* < 0.001), EDSS at the time of diagnosis (RR-MS, median [IQR] = 2 [1.0–2.5]; P-MS, median [IQR] = 3.5 [2.5–5.5]; *p* < 0.001), EDSS at first year after diagnosis (RR-MS, median [IQR] = 1 [1.0–2.0]; P-MS, median [IQR] = 5 [3.5–6.0]; *p* < 0.001) and EDSS at second year after diagnosis (RR-MS, median [IQR] = 1 [1.0–2.0]; P-MS, median [IQR] = 5.5 [3.5–6]; *p* < 0.001).

### 2.2. CSF Cytokine Levels Are Different in RR-MS, P-MS and Inflammatory Controls

**Figure 1:**
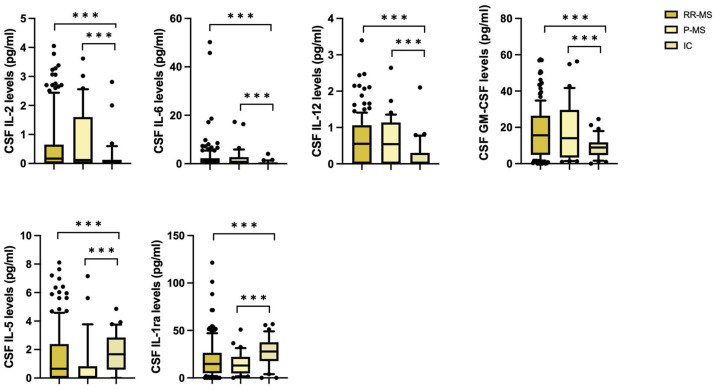
Differences in CSF cytokine concentrations between RR-MS and P-MS patients and inflammatory controls (non-parametric Kruskal–Wallis test). Legend: CSF (cerebrospinal fluid); RR-MS (relapsing–remitting MS); P-MS (progressive MS); IC (inflammatory controls); GM-CSF (granulomonocyte colony stimulating factor); IL-1ra (IL-1 receptor antagonist). Asterisks denote statistical significance (***, *p* < 0.005).

We first compared the CSF cytokine profile at the time of diagnosis in RR- and P-MS patients and inflammatory controls. The CSF levels of IL-2, IL-6, IL-12 and GM-CSF were significantly higher in patients than controls in a non-parametric Kruskal–Wallis test (IL-2 (median [IQR], RR-MS = 0.2 [0–0.7]; P-MS = 0.1 [0–1.6]; IC = 0.1 [0.0–0.1]; *p* < 0.005), IL-6 (median [IQR], RR-MS = 0.9 [0.3–2.3]; P-MS = 0.8 [0.1–2.7]; IC = 0.1 [0.0–0.5]; *p* < 0.005), IL-12 (median [IQR], RR-MS = 0.5 [0–1.1]; P-MS = 0.5 [0–1.1]; IC = 0.0 [0.0–0.3]; *p* < 0.005), GM-CSF (median [IQR], RR-MS = 15.6 [4.8–26.4]; P-MS = 14 [3.3–29.7]; IC = 8.9 [4.7–11.7]; *p* < 0.005)), while higher levels of IL-5 and IL-1ra were observed in controls (IL-5 (median [IQR], RR-MS = 0.65 [0–2.4]; P-MS = 0.1 [0–0.8]; IC = 1.7 [0.6–2.8]; *p* < 0.005), IL-1ra levels (median [IQR], RR-MS = 14.7 [4.9–26.4]; P-MS = 13.1 [4.7–22.2]; IC = 27.8 [17.7–37.6]; *p* < 0.005)) (Figure 1).

### 2.3. rs2069812IL-5 SNP Is Associated with Prospective Disease Activity in MS

To explore the effects of SNP rs2069812 on clinical characteristics, patients were divided in two groups according to the presence of the T-allele (CT/TT vs. CC) (Table 2). Inflammatory controls, divided according to rs2069812, present similar demographic characteristics in the two groups.

No significative associations were found between the presence of SNP rs2069812 and demographical characteristics in inflammatory controls. In MS patients, no significant differences were found in the two rs2069812 SNP groups in terms of clinical characteristics at the time of diagnosis. Conversely, a significant association was found with NEDA-3 assessed after a median follow-up of 36 months. In particular, the number of patients showing NEDA-3 status was significantly reduced in the T-carriers. Logistic regression confirmed a significant association between SNP and NEDA-3 status whilst also controlling for other characteristics including age, sex, disease duration, EDSS at diagnosis, presence of OCB at diagnosis and disease-modifying treatment (DMT) efficacy (IL-5 rs2069812 CT/TT, CC: S.E. 0.426, *p =* 0.007, 95% C.I. 1.364–7.248) (Table 3).

### 2.4. rs2069812 IL-5 SNP Influences Neuroinflammation in MS

**Figure 2:**
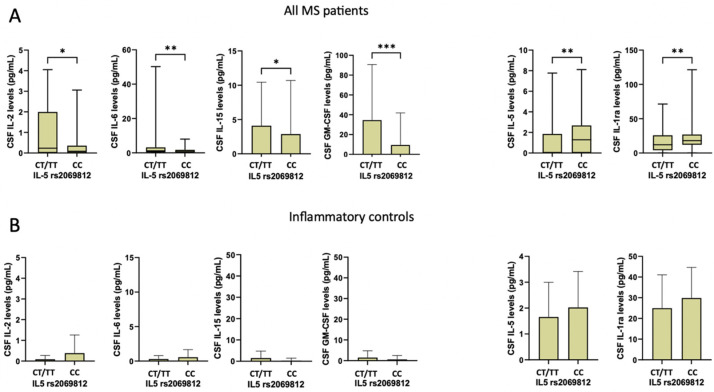
Differences in CSF cytokine concentrations according to rs2069812 IL-5 polymorphism in MS patients (**A**) and inflammatory controls (**B**) (non-parametric Mann–Whitney test). Legend: CSF (cerebrospinal fluid); GM-CSF (granulomonocyte colony stimulating factor); IL-1ra (IL-1 receptor antagonist). Asterisks denote statistical significance (*, *p* <0.05; **, *p* < 0.01; ***, *p* < 0.005).

We explored whether rs2069812 IL-5 polymorphism may affect CSF cytokine expression in our cohort of MS and control patients. In MS patients carrying the T-allele, higher levels of specific proinflammatory cytokines were found, including IL-2 (median [IQR], CT/TT = 0.2 [0.0–2.0]; CC = 0.1 [0.0–0.4]; Mann–Whitney test *p* = 0.015), IL-6 (median [IQR], CT/TT = 1.2 [0.4–3.2] vs. CC = 0.7 [0.1–1.7]; *p* = 0.007), IL-15 (median [IQR], CT/TT = 0.1 [0.0–9.5] vs. CC = 0.0 [0.0–0.1]; *p* = 0.019), and GM-CSF (median [IQR], CT/TT = 0.1 [0.0–0.6] vs. CC = 0.05 [0.0–0.1]; *p* < 0.001). In addition, the presence of the T-allele was associated with reduced expression of IL-5 (median [IQR], CT/TT = 0.03 [0.0–1.9] vs. CC = 1.28 [0.0–2.7]; *p* = 0.001) and IL-1ra (median [IQR], CT/TT = 12.1 [4.1–25.9] vs. CC = 18.1 [12.1–26.9]; *p* = 0.006) (Figure 2A). These differences were confirmed after controlling for multiple comparisons (corrected *p*: GM-CSF, *p* = 0.008; IL-6, *p =* 0.008; IL-2, *p =* 0.024; IL-1ra, *p =* 0.024; IL-15, *p =* 0.026; IL-5, *p =* 0.042).

In inflammatory controls, no significant differences were found in CSF cytokine levels between the CT/TT and CC groups (all *p* > 0.05) (Figure 2B).

**Figure 3:**
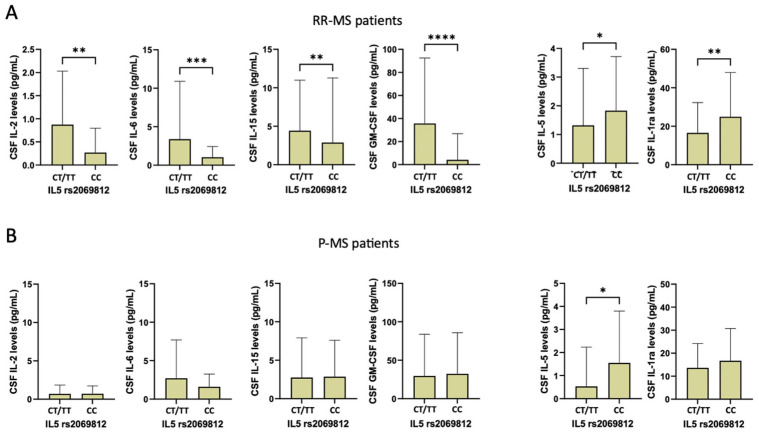
Differences in CSF cytokine concentrations according to rs2069812 IL-5 polymorphism in RR-MS (**A**) and P-MS patients (**B**) (non-parametric Mann–Whitney test). Legend: CSF (cerebrospinal fluid); GM-CSF (granulomonocyte colony stimulating factor); IL-1ra (IL-1 receptor antagonist). Asterisks denote statistical significance (*, *p* < 0.05; **, *p* < 0.01; ***, *p* < 0.005; ****, *p* < 0.001).

Finally, we examined the association between rs2069812 SNP and CSF inflammatory cytokines separately in RR-MS and P-MS patients. In patients with RR-MS, the presence of the T-allele was associated with increased levels of IL-2 (Mann–Whitney test, T-allele, median [IQR] = 0.3 [0.0–2.0] vs. C-allele, median [IQR] = 0.04 [0.0–0.3]; *p* = 0.005), IL-6 (T-allele, median [IQR] = 1.3 [0.4–3.3] vs. C-allele, median [IQR] = 0.6 [0.03–1.5]; *p* = 0.001), IL-15 (T-allele, median [IQR] = 0.1 [0.0–9.5] vs. C-allele, median [IQR] = 0.0 [0.0–0.1]; *p* = 0.008), and GM-CSF (T-allele, median [IQR] = 0.1 [0.0–97.9] vs. C-allele, median [IQR] = 0.0 [0.0–0.001]; *p* < 0.001), and with reduced levels of IL-5 (T-allele, median [IQR] = 0.02 [0.0–2.2] vs. C-allele, median [IQR] = 1.5 [0.0–2.9]; *p* = 0.016) and IL-1ra (T-allele, median [IQR] = 12.1 [4.3–26.4] vs. C-allele, median [IQR] = 18.5 [12.7–28.3]; *p* = 0.006) (Figure 3A).

In P-MS patients, a significant difference was found only for IL-5 CSF levels (T-allele, median [IQR] = 0.1 [0–0.23] vs. C-allele, median [IQR] = 0.6 [0.0–2.5]; *p* = 0.022) (Figure 3B).

## 3. Discussion

Previous studies suggested that genetic variability influencing the expression and function of specific inflammatory mediators may affect immune responses and clinical manifestations in different pathological conditions, including MS [10,15].

Here, we assessed the possible role of the rs2069812 SNP of the anti-inflammatory IL-5 in MS, exploring the associations with clinical characteristics and CSF inflammatory profiles at the time of diagnosis, and with prospective disease activity. We found that in RR-MS patients, the presence of the T-allele was associated with reduced expression of IL-5 and of IL-1ra, and with higher levels of several proinflammatory cytokines, including IL-2, IL-6, IL-15, and GM-CSF. Conversely in the P-MS group, a significant association was only found with reduced IL-5 concentrations. The inflammatory control group showed increased IL-5 and IL-1ra levels compared with MS patients. No significant associations between the rs2069812 SNP and CSF cytokines were found in the IC group.

These findings suggest that the rs2069812 SNP may influence CSF inflammatory profile in MS patients, modulating IL-5 expression and affecting the concentrations of various proinflammatory mediators.

IL-5 is considered an anti-inflammatory cytokine associated with increased expression of regulatory T-cells and reduced production of proinflammatory mediators [16]. Defective IL-5 expression may therefore exacerbate CSF inflammation, leading to dysregulated expression of various proinflammatory mediators. Notably, in experimental models of MS, and specifically in knock-out mice for the gene of IL-5, antigen-specific T-cells promoted the release of IFN-gamma and TNF-alfa, in the absence of anti-inflammatory cytokines such as IL-4 or IL-10, indicating a predominant Th1 environment following immunization [17].

Previous studies have evidenced that the rs2069812SNPs of IL-5 may also influence immune responses and clinical manifestations in other diseases. A study conducted by Chen and colleagues demonstrated a role of the T-allele of rs2069812 in increasing the risk of developing Non-Hodgkin lymphoma (NHL) [18], and another work showed the influence of this specific SNP in causing a decrease in IL-5 serum concentration, modifying Th1/Th2 response in terms of susceptibility for NHL [19]. It is noteworthy that rs2069812 seems to affect the immune systemic response in gastric [20] and hepatobiliary cancer [21] and in the development of alloantibodies against factor VIII during replacement therapy in hemophilia A [11]. Despite the lack of much evidence in this sense, some studies investigated the role of rs2069812 in systemic autoimmune diseases. A striking association between the onset and clinical presentation of Graves’ disease and the presence of C-allele for this polymorphism was found [22], and at the same time, the T-allele seems to be a risk factor for Hashimoto thyroiditis and a protective variant from the risk of developing Graves’ disease [23]. Other authors found that rs2069812, in its T-allele variant, could negatively influence clinical activity in seronegative spondylarthritis, as well as predicting pharmacological responses in ankylosing spondylitis [24].

In line with these data, we found that rs2069812 was associated with a lower probability of reaching the NEDA status in RR-MS patients carrying the T-allele. NEDA is a useful parameter that is largely used in clinical trials, and in clinical practice [25], it is defined as the absence of clinical relapses, EDSS disability progression sustained for 12 weeks, and new gadolinium-enhancing or new or enlarging T2 lesions in MRI scans [26].

Previous studies have shown that neuroinflammation represents one of the most relevant drivers in MS progression, associated with early neurodegeneration and axonal damage [27]. Increased CSF levels of proinflammatory cytokines have been associated with disease activity and disability [28,29]. Moreover, higher CSF concentrations of anti-inflammatory cytokines such as IL-10 have been associated with increased probability of reaching NEDA-3 status one year after the diagnosis [30].

Overall, our data suggest that the rs2069812 SNP can modulate IL-5 expression, influencing the CSF inflammatory milieu in MS. In line with a negative role of exacerbated neuroinflammation in the course of MS, we found that the same polymorphism was also associated with prospective disease activity.

This is the first study exploring the effect of SNPs of IL-5 in MS. However, some limitations of our study must be considered. Our data suggest that, even if the effect of SNPs on IL-5 levels is also evident in progressive MS patients, it seems to be clearer in RR-MS patients. This evidence could be explained at least in part by the fact that in our study, the number of patients diagnosed with P-MS was lower than those with RR-MS, reflecting the natural distribution of the progressive phenotype in MS patients. In this sense, results on the effects of neuroinflammatory mediators should be considered more cautiously. Moreover, the size of control group is smaller than the others and consists of patients affected by inflammatory diseases. Therefore, further studies are needed to better define differences between MS patients and non-inflammatory controls.

Understanding the role of IL-5 and the specific impact of the rs2069812 polymorphism in MS provides valuable insights into the disease pathogenesis and potential therapeutic targets. Modulating IL-5 activity could represent a novel approach to managing MS, either by dampening harmful inflammatory responses or by enhancing protective regulatory mechanisms. Further research is necessary to elucidate the precise mechanisms by which IL-5 and its genetic variants contribute to MS and to explore their potential in clinical applications [31].

Analyzing the interactions between IL-5, its genetic variants, and the broader immune system will enhance our understanding of MS and may lead to novel therapeutic strategies aimed at modulating immune responses to improve patient outcomes.

## 4. Materials and Methods

### 4.1. Patients

A group of 230 patients admitted at the Neurology Unit of Neuromed Hospital (Pozzilli, Italy) between 2016 and 2019 was included in the present study; 157 patients were diagnosed with relapsing–remitting MS (RR-MS) and 38 patients had progressive MS (P-MS), based on clinical, radiological and laboratory criteria [32]. The control group included 35 patients with MRI findings suggestive of inflammatory lesions that did not fulfill the diagnostic criteria for MS. The Ethics Committee of the Neuromed Research Institute approved the study (cod. 06-17) according to the Declaration of Helsinki, and all the participants provided written informed consent. For all the participants, at the time of diagnosis, a clinical examination and MRI brain and spinal cord assessments were performed. The following clinical characteristics were assessed in all MS patients: age, sex, expanded disability status score (EDSS), the presence of radiological disease activity and disease duration. Clinical and radiological follow-up data (EDSS, presence of clinical and radiological relapses) were available for 170 patients after 1 year and for 158 patients after 2 years.

No Evidence of Disease Activity-3 (NEDA-3) status, defined as a composite parameter of three related measures, which consist of an absence of clinical and radiological relapses and sustained disability progression at EDSS [33], was calculated for all RR-MS patients at the time of the last follow-up visit, with a median of three years.

### 4.2. SNP IL-5 rs2069812 Analysis

Patients were genotyped for IL-5 SNP rs2069812. A blood sample (200 μL) was collected at the time of diagnosis. The IL-5 gene region containing the rs2069812 polymorphism was amplified by a polymerase chain reaction with the TaqMan method performed using the ABI-Prism 7900HT Sequence Detection System (Applied Biosystems, Foster City, CA, USA) from 150 ng of genomic DNA in a final volume of 25 μL containing polymerase buffer, 1.5 mM MgCl2, 0.2 mM of each dNTP, 5 pmoles of each primer (sense: 5′–AACCTT-GACCCTGCAGAATG–3′; antisense: 5′–ATGGGATTGCACTTGGTCTC-3′) and 1 U of Taq DNA polymerase. Amplification reactions consisted of a first denaturation step of 95 °C for 5 min, followed by 35 cycles consisting of a denaturation step of 45 s at 95 °C, an annealing step of 45 s at 58 °C and an elongation step at 72 °C for 1 min, and by a final elongation step of 72 °C for 7 min. Sequencing analyses were performed with 10 ng of PCR products, purified with an Agenocourt AMPure PCR Purification kit (Agenocourt Bioscience Corporation, Beverly, MA, USA) in accordance with the manufacturer’s instructions, using 0.5 pmoles of the sequence primer (5′–AAACATCCGAGGACAAGGTG–3′) and the ABI PRISM BigDye Terminator v3.1 Ready Reaction Cycle Sequencing Kit (Applied Biosystem, Foster City, CA, USA). The sequencing reaction was performed according to the manufacturer’s instruction, and the sequencing product was purified using a CleanSEQ dye terminal removal kit (Agenocourt Bioscience Corporation Beverly, MA, USA) and run on the Applied Biosystems 3730 DNA Analyzer Instrument (Applied Biosystem, Foster City, CA, USA).

### 4.3. CSF Collection and Analysis

In a group of 216 patients (154 RR-MS, 37 P-MS, 25 inflammatory controls), a set 17 of proinflammatory and anti-inflammatory cytokines was dosed. CSF samples were withdrawn during hospitalization, at the time of diagnosis, by lumbar puncture (LP). No corticosteroids, immunomodulants or disease-modifying therapies were administered before LP. CSF samples were stored at −80 °C and later analyzed using a Bio-Plex multiplex cytokine assay (Bio-Rad Laboratories, Hercules, CA, USA). CSF cytokine levels were established according to a standard curve generated for the specific target and expressed as picograms/milliliter (pg/mL). Samples were analyzed in triplicate. The CSF cytokines analyzed included interleukin (IL)-1β, IL-2, IL-4, IL-5, IL-6, IL-7, IL-8, IL-9, IL-10, IL-12, IL-13, IL-15, tumor necrosis factor alpha (TNF-alfa), interferon-gamma (IFN-gamma), granulomonocyte colony stimulating factor (GM-CSF) and interleukin-1 receptor antagonist (IL-1ra).

### 4.4. MRI

All the patients underwent a 1.5T MRI scan, including the standard following sequences: dual-echo proton density, fluid-attenuated inversion recovery (FLAIR), T1-weighted spin-echo (SE), T2-weighted fast SE, and contrast-enhanced T1-weighted SE before and after intravenous gadolinium (Gd) infusion (0.2 mL/kg). Radiological activity at the diagnosis was characterized by the presence of Gd-enhancing (Gd+) lesions detected during hospitalization. All the sequences acquired were performed using a General Electric Signa HDXT MRI (GE Health Care, Chicago, IL, USA) equipped with an 8-channel head coil.

### 4.5. Statistical Analysis

A Shapiro–Wilk test was used to evaluate the normality distribution of continuous variables. Data were shown as the mean (standard deviation, SD) or median (interquartile range, IQR). Categorical variables were presented as absolute (n) and relative frequency (%). A Chi-square or, when necessary, Fisher exact test was employed to explore the association between categorical variables. Difference in continuous variables between the IL-5 rs2069812 groups was evaluated using a non-parametric Mann–Whitney test for a comparison between two samples and a Kruskal–Wallis test for three or more samples. A *p* value ≤ 0.05 was considered statistically significant. Box plots were employed to highlight statistically significant differences between groups. All the comparisons were performed using IBM SPSS Statistics version 13.0 for Windows/Mac (IBM Corp., Armonk, NY, USA).

Logistic regression was used to evaluate the association between the proportion of T- and C-alleles on the IL-5 rs2069812 polymorphism and other variables indicative of disease progression (EDSS at second and third year, NEDA-3 status) controlling for the effects of other clinical variables (age, sex, disease duration, OCB presence and radiological activity).

## Figures and Tables

**Table 1:** Clinical and demographic characteristics of MS patients and controls.

N (230)	RR-MS (157)	P-MS (38)	IC (35)
Age at LP (Median, IQR)	33.9 (25.7–44.0)	50.3 (45.6–56.8)	41.8 (32.3–49.7)
Sex, F/M (N, %)	108/49 (68.8)	19/38 (50)	23/12 (65.7)
Disease duration, months (Median, IQR)	5 (1.1–24.3)	24.6 (11.8–72.6)	-
Radiological activity (yes = 1, no = 0) (N/tot, %)	66/153 (43.1) *	10/34 (29.4) *	-
OCBs (yes = 1, no = 0) (N/tot, %)	120/154 (77.9) *	31/37 (83.8) *	-
EDSS at LP (Median, IQR)	2 (1.0–2.5)	3.5 (2.5–5.5)	-
EDSS at first year after LP (Median, IQR)	1 (1.0–2.0) *	5 (3.5–6.0) *	-
EDSS at second year after LP (Median, IQR)	1 (1.0–2.0) *	5.5 (3.5–6.0) *	-
NEDA-3 (yes = 1, no = 0) (N/tot, %)	54/142 (40.0) *	-	-

Legend: N (number); RR-MS (relapsing–remitting MS patients); P-MS (progressive MS patients); IC (inflammatory controls); LP (lumbar puncture); IQR (interquartile range); F (female); M (male); OCBs (oligoclonal bands); EDSS (expanded disability status scale); NEDA (no evidence of disease activity). * missing data, see Materials and Methods.

**Table 2:** Clinical characteristics of MS patients according to rs2069812 polymorphism.

N (195)	CT/TT Allele (115)	CC Allele (80)	*p*
Age at LP (Median, IQR)	38.8 (28.7–48.3)	36.2 (25.4–51.6)	0.604
Sex, F/M (N, %)	77/38 (67.0)	50/30 (62.5)	0.312
OCBs (yes = 1, no = 0) (N/tot, %)	93/115 (83) *	58/79 (73.4)	0.077
Disease duration, months (Median, IQR)	11.6 (1.3–33.8)	6.0 (1.7–36.0)	0.661
Radiological activity (yes = 1, no = 0) (N/tot, %)	43/109 (39.4) *	33/78 (42.3) *	0.404
EDSS at LP (Median, IQR)	2 (1.0–3.0)	2 (1–2.5)	0.214
EDSS at first year after LP (Median, IQR)	2 (1.0–3.0) *	1.5 (1–2) *	0.149
EDSS at second year after LP (Median, IQR)	2 (1.0–3.5) *	1 (1–2.5) *	0.120
NEDA-3 (yes = 1, no = 0) (N/tot, %)	24/81 (29.6) *	30/61 (49.2) *	**0.023**

Legend: N (number); LP (lumbar puncture); IQR (interquartile range); OCBs (oligoclonal bands); EDSS (expanded disability status scale); NEDA (no evidence of disease activity). Bold type denotes statistical significance. * missing data, see Materials and Methods.

**Table 3:** Logistic regression corrected for confounding factors in RR-MS patients between NEDA-3 and presence of C-allele for rs2069812 IL-5 polymorphism.

	B	S.E.	*p*	95% C.I.	Exp (B)
IL-5 rs2069812 groups (CT/TT, CC)	1.146	0.426	**0.007**	1.364–7.248	3.144
Sex (F = 1, M = 0)	0.408	0.455	0.370	0.616–3.668	1.503
Age	0.016	0.018	0.376	0.981–1.052	1.016
EDSS at LP	0.034	0.206	0.869	0.691–1.549	1.035
OCBs (1 = yes, 0 = no)	1.480	0.690	0.053	1.148–16.673	4.375
DMTs (high efficacy = 1, low efficacy = 0)	0.598	0.519	0.249	0.658–5.029	1.234
(Constant)	−3.463	1.093	0.002		0.031

Legend: LP (lumbar puncture); F (female); M (male); OCBs (oligoclonal bands); DMTs (disease-modifying treatments); EDSS (expanded disability status scale). Bold type denotes statistical significance.

## Data Availability

Anonymized datasets are available upon reasonable request to the corresponding author.

## Abbreviations

MS (multiple sclerosis); SNP (single-nucleotide polymorphism); CSF (cerebrospinal fluid); NEDA (no evidence of disease activity); EDSS (Expanded disability status scale); DMT (disease-modifying treatment); LP (lumbar puncture); OCB (oligoclonal band); MRI (magnetic resonance imaging); SD (standard deviation); IQR (interquartile range).
